# Retrospective Epidemiological Analysis of Influenza A Infections in a Single Hospital in Korea (2007–2024): Age, Sex, and Seasonal Patterns

**DOI:** 10.3390/pathogens14030282

**Published:** 2025-03-14

**Authors:** Jeong Su Han, Hyeong Ho Kim, Jae-Sik Jeon, Yoo Na Chung, Jae Kyung Kim

**Affiliations:** 1 Department of Biomedical Laboratory Science, College of Health Sciences, Dankook University, Cheonan-si 31116, Republic of Korea; jshan1162@naver.com (J.S.H.); hohyeongkim@dankook.ac.kr (H.H.K.); zenty87@naver.com (J.-S.J.); 2 Department of Medicine, College of Medicine, Dankook University, Cheonan-si 31116, Republic of Korea; nottinghum@naver.com

**Keywords:** chronic disease, health policy, influenza, real-time polymerase chain reaction, seasonal variation, vaccination

## Abstract

Influenza A is a respiratory virus that causes high infection rates and mortality worldwide, particularly affecting high-risk groups such as children, older adults, and individuals with chronic conditions. This retrospective study was conducted at a single tertiary hospital in Korea to analyze the epidemiological characteristics of influenza A infections from 2007 to 2024, focusing on age, sex, and seasonal variations. Using multiplex real-time PCR data from 23,284 individuals, we found that the overall positivity rate for influenza A was 5.6%, with seasonal fluctuations showing the highest rate in winter (14.0%) and the lowest in summer (0.5%). Age-based analysis revealed significantly higher positivity rates in older adults (7.9%) and adults (7.6%) than in children (5.0%) and infants (3.1%). No significant differences were observed in positivity rates between sexes (male: 5.43%, female: 5.76%, *p* = 0.428). These findings provide essential insights into the regional and seasonal patterns of influenza A, emphasizing the importance of targeted vaccination strategies, adaptive public health interventions, and continuous surveillance for effective prevention and outbreak control management.

## 1. Introduction

Influenza A is a respiratory virus that causes high infection rates and mortality worldwide, resulting in millions of hospitalizations and tens of thousands of deaths each year [1,2]. It poses a significant risk to vulnerable populations such as children, older adults, and individuals with chronic diseases [3,4,5], placing a considerable burden on global healthcare systems. Influenza A exhibits seasonal epidemic patterns [6], and the regular occurrence of these outbreaks presents a significant challenge to public health management [7]. These characteristics underscore the need for influenza vaccination policies and offer opportunities for more effective infection control strategies by understanding the virus’s mutations and seasonal trends.

Moreover, long-term data are valuable for analyzing regional epidemic patterns, seasonal variations, and the vulnerability of high-risk groups [8,9]. Gathering comprehensive data dating back to 2007 can offer valuable insights into understanding the trends in the spread of influenza A virus based on its mutations and seasonal characteristics. Such an approach is crucial in informing epidemic control and prevention strategies as well as in developing effective responses and policies for mitigating regional outbreaks and protecting the high-risk populations [10,11].

This study aimed to comprehensively analyze the epidemiological characteristics of influenza A infection using multiplex real-time PCR data from 2007 to 2024. Through this, the study sought to identify trends in regional epidemics and the infection patterns among high-risk groups such as children, older adults, and individuals with chronic diseases. The findings of the study are anticipated to establish the foundational data that can be leveraged for the development of future public health management and prevention strategies.

## 2. Materials and Methods

### 2.1. Study Design

This study was designed as a retrospective observational study using data from molecular identification tests for respiratory viruses conducted at Dankook University Hospital (Cheonan-si, South Korea) during the study period. The study data include results from multiplex real-time PCR tests related to influenza A collected from 2007 to 2024.

### 2.2. Data Collection

This study included all individuals who underwent multiplex real-time PCR testing for respiratory viruses at Dankook University Hospital from January 2007 to December 2024. Patients were included regardless of symptom severity or clinical suspicion. Cases with missing essential data (e.g., age, sex, or test date) were excluded from the analysis to maintain data integrity. The distribution of study participants by age group and sex is shown in Table 1.

### 2.3. RNA Extraction and Real-Time PCR

Nucleic acid was extracted from nasal swabs using the QIAamp Viral RNA Mini Kit (Qiagen, Hilden, Germany), strictly following the manufacturer’s protocol. The extracted nucleic acid was analyzed using a multiplex real-time PCR kit (LG Life Sciences, Changwon-si, South Korea), which includes specific primers and probes for detecting the influenza A gene. PCR reactions were performed using the SLAN real-time PCR equipment (LG Life Sciences, Changwon-si, South Korea) following the manufacturer’s recommended conditions. The interpretation of PCR results was based on the cycle threshold (CT) values provided by the manufacturer. Positive results were defined as those with a CT ≤ 40, which was used as the criterion to reliably confirm the presence of the virus.

### 2.4. Data Preprocessing

Missing data were excluded from the analysis. Age groups were categorized based on The International Council for Harmonisation of Technical Requirements for Pharmaceuticals for Human Use (ICH) age classification guidelines used in South Korea: infant (0 years); child (1–19 years); adult (20–64 years); and older adults (65 years and above). Data were categorized by sex and test date, and seasonal and annual variables were created for analysis.

### 2.5. Data Analysis

Seasonal incidence frequencies were calculated, and data analysis of key variables was performed using Excel 2024. The expected positivity rates for each age group and season were determined by calculating the observed influenza A positivity rate within each subgroup and multiplying it by the total number of individuals in that group, while the expected number of negative cases was obtained by subtracting the expected positive cases from the total cases. To minimize selective testing bias, broad inclusion criteria encompassing all RT-PCR-confirmed influenza A cases were applied. Additionally, a longitudinal data analysis covering 18 years (2007–2024) was conducted to account for temporal variations in testing protocols and seasonal effects. Sex- and age-based analyses were performed using Z-tests and chi-square tests in SPSS software (version 17.0, SPSS Inc., Chicago, IL, USA), allowing comparisons of positivity rates across different demographic and seasonal subgroups and mitigating the impact of sample distribution differences.

### 2.6. Ethical Considerations

This study was approved by the Institutional Review Board of Dankook University (IRB No. [DKU 2023-03-004-005]). Personal identifying information was excluded from this study and data were anonymized before analysis. Informed consent from participants was waived owing to the nature of the study.

## 3. Results

### 3.1. Annual Trends of Incidence

**Figure 1:**
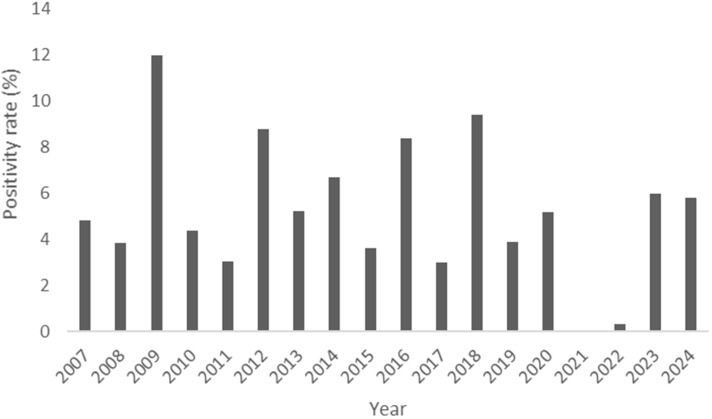
Influenza A positivity rate from 2007 to 2024.

The annual positivity rate of influenza A from 2007 to 2024 was analyzed (Figure 1). The data over 18 years showed significant fluctuations in the influenza A infection rate, with a sharp increase observed in 2009. From 2010 to 2020, the positivity rate remained relatively stable. However, in 2021 and 2022, a sharp decline was observed. During this period, the transmission of influenza A was significantly suppressed, leading to a near-zero positivity rate. The positivity rate increased again in 2023.

### 3.2. Seasonal Patterns

**Figure 2:**
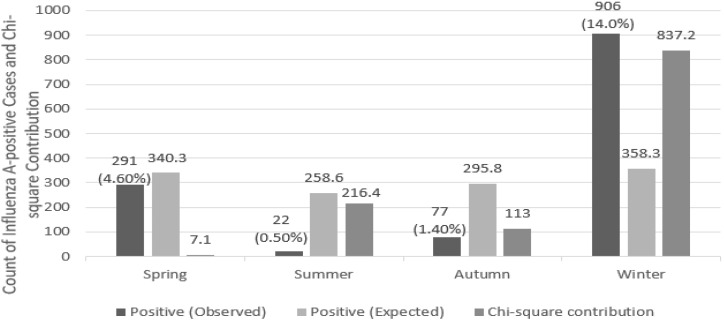
Seasonal influenza A positivity rates and contributions from 2007 to 2024.

A total of 23,284 specimens, collected from 2007 to 2024, were included in this study. Statistically significant seasonal differences in the positivity rate of influenza A were examined by analyzing data from 2007 to 2024 across four seasons: spring (March–May), summer (June–August), fall (September–November), and winter (December–February). The observed and expected positivity rates, as well as their chi-square contributions, are shown in Table 2 and Figure 2.

The chi-square test revealed that the critical value was 7.815, with χ^2^(3) = 1173.7, which greatly exceeded the threshold, indicating a significant difference in the influenza A positivity rate by season (*p* < 0.05). When the positivity rates are examined by season, the winter season had the highest positivity rate, while the summer season had the lowest positivity rate. In winter, the proportion of positive cases was highest across the entire sample, whereas in summer, the positivity rate was very low, indicating a significant difference in the pattern of occurrence of influenza A (*p* < 0.001).

### 3.3. Sex-Based Analysis

**Figure 3:**
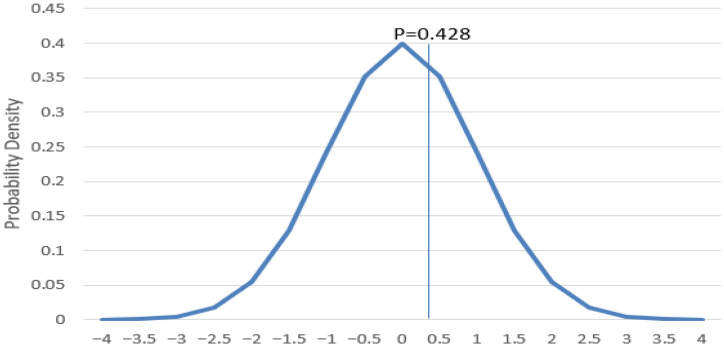
Distribution of *p*-values for influenza A positivity rates by sex from 2007 to 2024.

To analyze statistically significant differences in influenza A positivity rates between men and women, data were collected from 13,961 men, among which 759 tested positive (positivity rate: 5.43%), and 9323 women, among which 537 tested positive (positivity rate: 5.76%). The distribution of *p*-values for influenza A positivity rates by sex is shown in Figure 3. Chi-square analysis revealed no statistically significant differences in influenza A positivity rates between sexes (*p* = 0.428).

### 3.4. Age-Based Analysis

**Figure 4:**
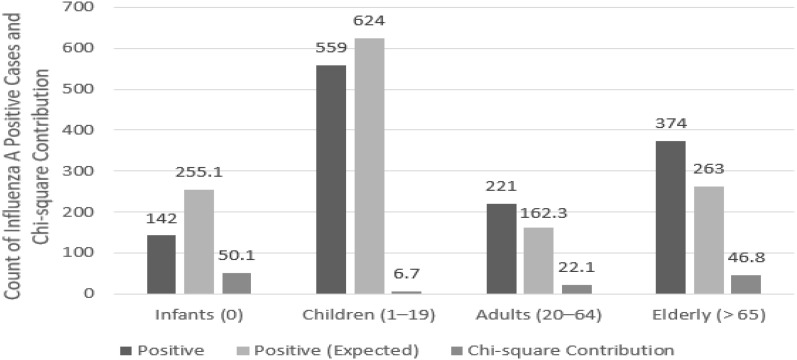
Influenza A positivity rates and chi-square contributions by age group from 2007 to 2024.

The total numbers of individuals and positive cases for each age group are shown in Table 3. The influenza A positivity rate tended to increase with age. To test the statistical significance between these data, the positivity rates of influenza A across different age groups were compared and analyzed. To compare the infection rates by age group, the expected values for both positive and negative cases in each age group were calculated. The overall infection rate was found to be 0.05%, and based on this, the expected values for positive and negative cases were derived for each age group (Table 4). Chi-square analysis was performed based on the expected values for positive and negative cases by age group. The influenza A positivity rates and chi-square contributions by age group are shown in Figure 4. The analysis results show that χ^2^(3) = 124.8, which is greater than the critical value of 7.815, indicating a statistically significant difference in influenza A infection rates across different age groups (*p* < 0.001). Among these, higher infection rates were observed in the older adults and adult groups.

## 4. Discussion

### 4.1. Annual Trends of Incidence

The significant increase in 2009 can be attributed to a large-scale H1N1 epidemic [12,13]. Despite the relative stability of positivity rates between 2010 and 2020, continuous public health measures remained necessary to prevent the spread of influenza. The sharp decline in 2021 and 2022 was likely influenced by social changes brought about by the COVID-19 pandemic, including large-scale quarantine measures, mask-wearing, and hand hygiene, which played a key role in reducing influenza A transmission [14,15,16]. The increase in positivity rates from 2023 onward suggests a gradual return to normal life, indicating that influenza A positivity rate trends are influenced not only by time but also by ecological, social, and policy factors. This highlights the importance of a multifaceted approach in predicting and responding to future infectious disease outbreaks. Moving forward, influenza vaccination will continue to play a critical role, and future changes in positivity rates will depend on various health policies and prevention strategies. The fluctuations in influenza A positivity rates are closely linked to public health policies, infectious disease outbreaks, and social behavior changes. Understanding these trends will enable the development of more effective influenza prevention strategies, strengthening preventive measures and enhancing preparedness for future pandemics.

### 4.2. Seasonal Patterns

The seasonal epidemic cycle of influenza A has been linked to seasonal factors, with higher infection rates observed in winter and fall [17]. This difference in infection rates can be attributed to seasonal changes, such as temperature and humidity, which influence the transmission and survival of the influenza virus. Additionally, previous studies suggest that influenza viruses remain stable and viable for longer durations under low temperature and humidity, which may further contribute to the seasonal increase in detection rates. Future studies should consider viral stability as a key factor in understanding seasonal influenza transmission dynamics. In winter, the higher density of people indoors and lower humidity increase the survival rate of the virus, and the closer contact between individuals makes it easier for the infection to spread [18,19].

Additionally, seasonal variations can affect people’s immunity. In winter, people spend more time indoors and are less exposed to sunlight, which can lead to a deficiency in vitamin D and a reduction in immune function [20,21]. These factors can further accentuate the seasonal differences in influenza A infection rates [22].

The results show that the prevalence of influenza A is influenced by seasonal factors, suggesting the need to consider seasonal characteristics in future prevention and management strategies. Focused preventive measures should be tailored to specific seasons, such as increasing vaccination rates during winter and fall when influenza infection rates tend to spike [23,24]. Furthermore, while this study analyzed data from 2007 to 2024, long-term monitoring could provide a clearer understanding of seasonal fluctuations in infection rates. Comparing these results with those from other regions and countries and evaluating the impact of other social and environmental factors on infection rates would also be important.

### 4.3. Sex-Based Analysis

The results of sex-based analysis suggest that sex does not have a significant impact on influenza A infection, implying that sex-specific differentiation in prevention and treatment strategies may not be necessary. However, while this study found no significant sex-based differences, further research is needed to account for additional biological, behavioral, and social factors that may influence influenza susceptibility. Future studies should consider hormonal influences, healthcare-seeking behavior, occupational exposure, and vaccination status to determine whether more subtle sex-based differences exist in influenza A infection rates.

### 4.4. Age-Based Analysis

The results suggest that adults and older individuals have higher influenza A infection rates, highlighting the need for targeted prevention and treatment strategies. Older adults are more vulnerable to infections due to weaker immune systems [25,26], making tailored and detailed preventive measures and treatment approaches essential for this demographic. For adults aged 20–64 years, the relatively high influenza A positivity rate is likely influenced by increased social interactions, occupational exposure, and frequent healthcare visits rather than weakened immune responses. These factors may contribute to higher transmission risk in this age group, underscoring the need for preventive measures tailored to their lifestyle and exposure patterns. However, this study did not involve a detailed subgroup analysis within these high-risk populations, such as stratifying based on comorbidities, vaccination status, or hospitalization rates. Future research should incorporate these factors to provide a more refined understanding of influenza A susceptibility and disease severity among high-risk individuals.

The infection rates in children and infants were relatively low. Children and infants are mostly protected within the home environment and are exposed to fewer external factors, which could reduce the likelihood of being exposed to infectious agents. However, the lower influenza A positivity rate in children may not be solely due to reduced exposure. Studies have shown that respiratory syncytial virus exhibits an exceptionally high positivity rate (over 90%) in infants, whereas influenza A virus is more commonly detected in older age groups. This suggests that influenza A virus may have age-specific immunological and epidemiological characteristics that influence infection patterns. Additionally, children are frequently exposed to multiple respiratory viruses, which may create a competitive infection environment, reducing the likelihood of testing positive for influenza A. Given these factors, it is crucial to consider not only exposure but also age-dependent susceptibility, immune response variations, and viral interactions when interpreting the lower influenza A positivity rate in children. Future research should further investigate the interplay between different respiratory viruses and the mechanisms underlying age-related differences in influenza A susceptibility and transmission.

In contrast, adults and older adults tend to have more social interactions and are more likely to visit healthcare facilities, increasing their chances of exposure to various infectious agents [27]. From a vaccination perspective, children and infants typically receive regular vaccinations, which provide immunity against influenza A. These vaccinations play a crucial role in preventing infections [28]. However, older individuals often have lower vaccination rates, and even if they receive the vaccine, their weakened immune systems may result in slower antibody production, leaving them more susceptible to infections [29,30,31].

This study emphasizes the importance of considering age when developing prevention and management strategies for influenza A. Focused prevention and treatment efforts for high-risk groups are necessary, which could contribute to reducing overall infection rates. However, relying on a single technique such as real-time PCR for diagnosing influenza A may introduce errors due to its limitations, highlighting the need for additional research to improve diagnostic accuracy. In this study, we analyzed the positivity rates of influenza A virus but focused on the overall detection of influenza A infections rather than distinguishing specific subtypes such as H3N2 or H1N1. Identifying subtypes could provide a more in-depth understanding of seasonal influenza trends. Although this study did not include subtype classification, future research should consider this aspect to conduct a more detailed epidemiological analysis of influenza A infections.

Additionally, while we analyzed influenza A positivity rates separately by age group and sex, we did not perform subgroup analysis comparing sex differences within the same age group or age differences within the same sex group. Future studies should incorporate such interaction analyses to provide deeper insights into the combined effects of age and sex on influenza transmission. This study primarily focused on analyzing influenza A virus positivity rates across different age and sex groups. However, individual vaccination records were not included in our dataset, which limited direct evaluation of the impact of immunization on influenza positivity rates. While previous studies have shown that vaccination plays a crucial role in reducing the influenza burden, our findings provide important demographic insights into the distribution of influenza A infections. Future studies should consider integrating vaccination history to further elucidate its influence on influenza transmission and infection rates. Furthermore, analyzing other factors (such as living environments and social factors) that influence age-specific infection rate differences is essential. This will provide valuable foundational data to develop more effective prevention and management strategies for influenza A in the future. Based on our findings, we propose strengthening vaccination programs for high-risk groups, implementing seasonal prevention measures such as improving indoor ventilation, humidity control, and public awareness campaigns, and enhancing real-time influenza surveillance systems to improve early detection and outbreak management. These strategies may contribute to the development of more effective influenza control policies and help reduce the disease burden.

## 5. Conclusions

This study provides novel insights into the long-term epidemiological trends of influenza A from 2007 to 2024, highlighting key seasonal and demographic variations. Our findings reveal that despite frequent exposure to respiratory viruses, children exhibit lower influenza A positivity rates, suggesting the influence of age-specific immune responses and viral interactions. Public health interventions during the COVID-19 pandemic significantly suppressed influenza A transmission, demonstrating the impact of large-scale health measures on respiratory virus dynamics. Influenza A positivity rates are shaped not only by seasonal factors but also by social behaviors and public health policies, emphasizing the need for continuous monitoring and adaptable prevention strategies. These findings contribute to a deeper understanding of influenza A transmission and underscore the importance of targeted vaccination campaigns, adaptive public health policies, and ongoing surveillance systems in mitigating future outbreaks. Future studies should further investigate the interplay between immunological, environmental, and social factors influencing influenza A dynamics.

## Figures and Tables

**Table 1:** Distribution of study participants (*n* = 23,284) by age group and sex.

Age Group	Male (*n*)	Female (*n*)	Total (*n*)
Infants (0)	2709	1844	4556
Children (1–19)	6341	4796	11,137
Adults (20–64)	1872	1027	2899
Older adults (>65)	3036	1656	4692
Total	13,958	9323	23,284

**Table 2:** Observed and expected influenza A positivity rates from 2007 to 2024 by season.

Season	Positive (Observed)	Positive (Expected)	Negative (Observed)	Negative (Expected)	Positivity Rate (%)
Spring	291 (4.6%)	340.3	6100 (95.4%)	6050.7	4.6%
Summer	22 (0.5%)	258.6	4788 (99.5%)	4551.4	0.5%
Autumn	77 (1.4%)	295.8	5530 (98.6%)	5311.2	1.4%
Winter	906 (14.0%)	358.3	5570 (86.0%)	6117.7	14.0%

**Table 3:** Influenza A positivity rates by age group from 2007 to 2024.

Age Group	Total Individuals	Positive	Negative	Positivity Rate (%)
Infants (0)	4556	142	4414	3.1
Children (1–19)	11,137	559	10,578	5.0
Adults (20–64)	2899	221	2678	7.6
Older adults (>65)	4692	374	4318	7.9

**Table 4:** Expected values for positive and negative cases by age group between 2007 and 2024.

Age Group	Positive (Expected)	Negative (Expected)
Infants (0 years)	255.1	4301
Children (1–19 years)	624	10,513
Adults (20–64 years)	162.3	2737
Older adults (65 years and above)	263	4429

## Data Availability

The authors confirm that the data supporting the findings of this study are available within the article.

## Abbreviation

The following abbreviation is used in this manuscript:

CT	Cycle threshold
